# Biological evaluations of decellularized extracellular matrix collagen microparticles prepared based on plant enzymes and aqueous two-phase method

**DOI:** 10.1093/rb/rbab002

**Published:** 2021-03-13

**Authors:** YaWen Liu, Ching-Cheng Huang, YuanYuan Wang, Jun Xu, GuoDing Wang, XinPeng Bai

**Affiliations:** Engineering Research Center of Utilization of Tropical Polysaccharide Resources, College of Food Science and Technology, Hainan University, Haikou, Hainan 570000, China; PARSD Biomedical Material Research Center (PBMRC), Changzhou, Jiangsu 213000, China; Engineering Research Center of Utilization of Tropical Polysaccharide Resources, College of Food Science and Technology, Hainan University, Haikou, Hainan 570000, China; PARSD Biomedical Material Research Center (PBMRC), Changzhou, Jiangsu 213000, China; Department of Biomedical Engineering, Ming-Chuan University, Taoyuan 320-33, Taiwan; Engineering Research Center of Utilization of Tropical Polysaccharide Resources, College of Food Science and Technology, Hainan University, Haikou, Hainan 570000, China; Engineering Research Center of Utilization of Tropical Polysaccharide Resources, College of Food Science and Technology, Hainan University, Haikou, Hainan 570000, China; PARSD Biomedical Material Research Center (PBMRC), Changzhou, Jiangsu 213000, China; Engineering Research Center of Utilization of Tropical Polysaccharide Resources, College of Food Science and Technology, Hainan University, Haikou, Hainan 570000, China; Engineering Research Center of Utilization of Tropical Polysaccharide Resources, College of Food Science and Technology, Hainan University, Haikou, Hainan 570000, China; PARSD Biomedical Material Research Center (PBMRC), Changzhou, Jiangsu 213000, China

**Keywords:** acellular dermal matrix, papain, *Carica papaya* lipase, aqueous two-phase extraction

## Abstract

For patients with extensive full-thickness burns who do not have sufficient autologous split-thickness skin for skin grafts, the application of biological skin substitutes may be considered. The aim of this study was to find an optimal new type method for the production of a biovital skin substitute based on acellular dermal matrix (ADM) and preclinical evaluations. In this work, 25 methods of ADM production were assessed. The proposed methods are based on the use of the following enzymes: papain, *Carica papaya* lipase (CPL), and purification using a polymer/salt aqueous two-phase system. The obtained ADM samples were characterized via scanning electron microscopy (SEM), porosity measurement and water vapor transmission test. Results showed that the collagen bundles of ADM microparticles were intact and orderly. Through differential scanning calorimetry (DSC), thermo gravimetric analysis (TGA) and biocompatibility tests, the results indicated that the proportion of papain and CPL was the same and 5 h processing time are the optimum conditions for ADM preparation and the material showed good biocompatibility. Our results suggested that the potential of developing this kind of decellularization process to manufacture ADM scaffolds for clinical application.

## Introduction

Skin is the largest organ and the first line of defense against bacterial infection, mechanical forces, fluid imbalance and thermal dysregulation in the human body [1, 2]. The serious injury caused by inflammation, ulcers, extensive burns, wounds, postoperative holes and other large areas of skin defects trigger the epidermis and dermis or even the subcutaneous tissue changes, such as metabolic disorders, infections, decreased immune function, high metabolic reactions and water and electrolyte disorders, which is disastrous for patients in terms of human life, affliction, disability and unaesthetic scars [3, 4]. Therefore, covering the wound and restoring skin barrier function are important. The current commonly used clinical treatment is autologous skin grafting. However, insufficient donor skin and damaged areas extremely restrict its application [5, 6]. Therefore, biocompatible materials become more attractive and have gradually replaced self-healthy skin, which can repair wounds, induce the orderly growth of host fibroblasts and capillaries, make the arrangement of collagen fibers more regular, and reduce local inflammation. Relevant studies showed that the acellular dermal matrix (ADM) derived from heterogeneous skin has great application prospects in the field of burns, trauma and plastic surgery; it has been applied in clinical tissue repair. ADM samples have a compact collagen structure, and the dermal matrix has a basement membrane component, which removes fatty cells, fibroblasts, vascular endothelial cells and other cellular components from the corium [7]. They provide a scaffold for proliferation and advantageously combine with the edge of the wound site [8].

At present, we have three traditional methods for ADM preparation: enzyme-decontamination method [9], acid–base method (sodium hydroxide [10, 11], calcium oxide and peracetic acid) and balanced salt solution method [12, 13]. However, during decellularization, a balance between the complete clearance of cellular residues and the retention of ADM components is difficult to maintain. Chemical decellularization is highly effective in removing cell residues typically but result in disruptive effects on the tissue structure of the matrix material and the integrity of the outer surface of the tissue. Moreover, washing the solvent thoroughly can be difficult. The disadvantages of a balanced salt solution is that the processing time is too long, the operation can easily cause pollution, and the process is complex, which will reduce the survival rate of composite wound grafts [14]. The enzymatic method, due to its gentle action, high efficiency and ability to retain the complete collagen structure, has gradually become a common method in the field of decellularization. Among the commonly used enzymes are trypsin, pepsin and neutral protease, which all originate from animals and have a number of disadvantages and limitations. Therefore, in this work, we developed a new method for removing cells from tissues by using the enzymes of plants.

Papaya is a tropical plant; unripe fruits have a milky thixotropic latex, which contains about 15% of dry matter, 85% water and a large variety of hydrolytic enzymes, mainly proteases [15, 16]. Papain has a wide range of sources and features simple preparation, high activity, non-toxicity and a relatively low price, so it is widely used. Papain is a kind of sulfhydryl-containing endonuclease with protease and esterase activity, and it has strong hydrolysis ability on proteins, polypeptides, esters and amides of animals and plants [17]. *Carica papaya* lipase (CPL) is tightly attached to the dry matter present in the *C. papaya* latex and is insoluble in water. Traditionally, CPL has been considered a naturally immobilized biocatalyst. CPL is a selective enzyme for triglyceride hydrolysis of short-chain fatty acids, so it shows the maximum hydrolysis activity for C4–C8 fatty acids. Simultaneously, experiments have demonstrated the sn-3 selectivity of CPL [18, 19]. Papain and CPL are relatively efficient in proteolytic enzymes and lipases among plants.

Aqueous two-phase extraction (ATPE) has been recognized as a promising new biochemical separation technology that has emerged in recent years; it uses the difference in the distribution of substances between two waters to separate and purify substances [20, 21]. The major advantages of ATPE are high capacity, mild extraction conditions, high biological compatibility, biocompatible environment, low interfacial tension of phases, low reaction time and low energy [22]. ATPE has been applied to the purification of various proteins and enzymes, but very limited information has been reported about the use of ATPE in ADM sample purification processes. The simplicity of the method and the low cost of phase forming materials enable the use of appropriate scale-up techniques for large-scale protein purification. Meanwhile, this technique has the potential to realize the recycling of phase substances and product purification in a single step.

In this work, we demonstrated a novel decellularization method of using plant enzymes (papain; CPL) and purification by using an aqueous two-phase system. The structure, morphology and mechanical properties of the fabricated scaffolds are analyzed through infrared spectroscopy, differential scanning calorimetry (DSC), thermogravimetric analysis (TGA), scanning electron microscopy (SEM) and biocompatibility experiments.

## Materials and methods

### Chemicals

Polyethylene glycol (PEG; MW 6000) was procured from Shanghai Macklin Biochemical Co., Ltd. Sulfuric acid salts were obtained from Xilong Science Co., Ltd. Papain and CPL were supplied by Nan Ning Doing-Higherbio-Techno., Ltd. Cell counting kit-8 (CCK-8) was produced by Guangzhou Saiguo Biotech Co., Ltd. Pig skin was supplied by Parsd Pharmaceutical Technology Co., Ltd. Rabbit blood was supplied by Hainan Medical University. Mouse fibroblasts (L-929 cell lines) were purchased from the Beijing Cell Bank of the Chinese Academy of Science. All the reagents used were of analytical grade.

GL-20G high-speed refrigerated centrifuge (Shanghai Anting Scientific Instrument Co., Ltd), FDW-200 vacuum freeze drier (Physical and chemical instruments, Tokyo), Spectra Max M5 multi-function microplate reader (Molecular Devices, USA), and F-280 fluorospectro photometer (Tianjin Gangdong Sci. &Tech. Co., Ltd) were employed in this work.

### Enzyme treatment

A blade was elevated approximately 1 mm from the surface of the platform to achieve a cut through the dermis. About 1 U/mL CPL [16, 23] and 1 U/mL papain were prepared. According to the Table 1, the ratio of material to liquid was 1:20. The samples were added with 5% Triton X-100 and mixed with an automatic mixer for 30 min, 1 h, 3 h, 5 h and 7 h. Excess liquid was discarded, and the samples were washed twice with phosphate buffer saline (PBS, pH 7.4) and ultra-pure water by ultrasound treatment.

**Table 1.** Different conditions for ADM preparation

Study group	Enzyme ratio	PH	Hour
ADM-1	Papain 0% CPL 100%	6.2	30 min
ADM-2		6.5	1 h
ADM-3		6.3	3 h
ADM-4		6.6	5 h
ADM-5		6.8	7 h
ADM-6	Papain 25% CPL 75%	6.2	30 min
ADM-7		6.5	1 h
ADM-8		6.3	3 h
ADM-9		6.6	5 h
ADM-10		6.8	7 h
ADM-11	Papain 50% CPL 50%	6.2	30 min
ADM-12		6.5	1 h
ADM-13		6.3	3 h
ADM-14		6.6	5 h
ADM-15		6.8	7 h
ADM-16	Papain 75% CPL 25%	6.2	30 min
ADM-17		6.5	1 h
ADM-18		6.3	3 h
ADM-19		6.6	5 h
ADM-20		6.8	7 h
ADM-21	Papain 100% CPL 0%	6.2	30 min
ADM-22		6.5	1 h
ADM-23		6.3	3 h
ADM-24		6.6	5 h
ADM-25		6.8	7 h

### Aqueous two-phase purification

A predetermined ratio of PEG 6000 and ammonium sulfate (55:45) was weighed, and 5 mg of ADM sample was added to reach a total weight of 100% (w/w). The purification experiments were conducted in ATPE composed of 40 wt% PEG6000 + 35 wt% (NH_4_)_2_SO_4_. The contents were mixed thoroughly using an electric stirrer to reach equilibrium, and phase separation was carried out for 4–5 h. All experiments were carried out at 25°C, and the pH value of the system was between 5 and 5.5. All the samples were stored at −40°C for 24 h and dried by a vacuum freeze dryer. The ADM pieces were ground to microparticles by using a cutting grinder.

## Mechanical properties of obtained samples

### Porosity assay

A 25 ml volumetric flask was added with ethanol (100%) at 20°C; the mass was weighed and recorded as M_1_. M_0_ was recorded as the weight of the ADM sample. The sample was completely immersed in absolute ethanol, sealed, and allowed to stand at 20°C for 24 h. Absolute ethanol above the graduation line in the volumetric flask was sucked off, and the weight was recorded as M_2_. The sample was removed and the weight was recorded as M_3_. Porosity was calculated with Equation (1) as follows:

(1) $$K(\%)=\frac{M_{2}-M_{3}-M_{0}}{M_{1}-M_{3}}\times 100\%$$

### Water vapor transmission rate assay

Test tubes with a diameter of 1 cm were filled with saline (0.9%), and the film was in contact with the liquid surface. No intermediate gaps were left, and sides along the tube were sealed and weighed. The system was placed in the dryer at 36°C ± 1°C and weighed after 24 h. The water vapor transmission rate was calculated as follows:

(2) $$W_{r}=(M_{0}-M_{1})/S$$

where W_r_: Water vapor transmission rate (g·m ^−2^·24h^−1^); M_0_: Original weight (g); M_1_: Weight after 24 h (g) and S: Surface area (m^2^).

### ADM samples characterization by SEM

SEM was carried out to show the collagen shape and structure of scaffolds. Prior to imaging by SEM, the surface was sputtered for 60 s with gold to increase conductivity, and the surface microstructure was observed by a tungsten filament SEM.

### ADM characterization by DSC

DSC analyses of ADM collagen microparticles were performed at low heating speed of 5 or 10°C·min^−1^. About 3–5 mg of ADM samples was placed in a hermetic aluminum pan. Dried nitrogen was used as a carrier gas at a rate of 20 ml·min^−1^. Temperature ranged from 20 to 150°C. T_d_ was recognized as the temperature at the maximum peak.

### Determination of ADM samples by TGA

TGA analysis is a technique for measuring the relationship between the mass of a substance and the temperature under a temperature control program. TGA was carried out from room temperature to 550°C under nitrogen atmosphere. Samples of approximately 3–5 mg were placed in an alumina pot at a heating rate of 20°C·min^−1^. The two weight loss steps were reported in TGA analysis: the first stage was assumed as moisture loss; the second one was assigned to the degradation of proteins.

### Fourier Transform infrared spectroscopy (FTIR) assay of ADM samples

To confirm the degree of purification and the composition of the ADM samples, FTIR was employed. Wavenumbers and FTIR spectra of ADM samples were observed at a range of 4000–400 cm^−1^.

### DNA quantification

About 5 mg of ADM collagen microparticles was digested using proteinase K solution for 4–6 h at 56°C. Digested samples were added the same volume of phenol water and centrifuged at 18 000 g for 10 min at room temperature. The supernatant was transferred to a new tube and added with isoamyl alcohol and trichloromethane (1:24). The above operation was repeated three times. Finally, the supernatant was mixed in alcohol, and the precipitate was collected. The precipitate was dissolved in DE buffer, and stored at −20°C. The solution of each group was analyzed by PicoGreen DNA fluorescence staining. Fluorescence was measured using a fluorescence spectrophotometer F-280 (excitation: 480 nm, emission: 520 nm), and values were compared with a DNA standard curve.

### Hemolysis assay

About 8 ml of fresh blood was drawn from healthy rabbit by employing venipuncture into tubes containing potassium oxalate anticoagulant, followed by dilution with 10 ml of 0.9% saline solution. About 2 g of ADM microparticles was placed into a tube containing 10 ml of 0.9% saline solution and then incubated at 37°C for 30 min. Subsequently, 0.2 ml of diluted blood was added into the test tube and further incubated at 37°C for 60 min. The 0.2 ml blood sample was incubated in 10 ml of distilled water and 0.9% saline solution as negative control. After centrifugation for 5 min at 3000 rpm, the absorbance of the supernatant at 540 nm was collected. The hemolysis percentage (HP) was calculated using Eq. (3):

(3) $$HP(\%)=\frac{\left(Dt-Dnc\right)}{\left(Dpc-Dnc\right)}\times 100\%$$

where Dt, Dpc and Dnc are the absorbance of the test samples, positive and negative controls, respectively.

### Cytotoxicity studies

The CCK-8 assay was performed to investigate the cytotoxicity of ADM microparticles. Mouse fibroblasts (L-929 cell lines) were cultivated in Dulbecco’s modified eagle medium (DMEM) with penicillin–streptomycin and 10% fetal bovine serum under a 37°C humidified incubator containing 5% CO_2_ and harvested in the log phase growth period for cytotoxicity analysis. The sample extract solution was extracted with DMEM + 10% fetal bovine serum cell culture medium and stored at 4°C. Cells were seeded onto the wells of a 96-well plate at a density of 1 × 10^3^ per mL, and then incubated for 72 h in an air atmosphere with 5% CO_2_. The viability of cultured fibroblasts was monitored after 72 h of culture using the colorimetric CCK-8 assay, and the optical density (OD) was measured at 450 nm using a microplate reader. No substance was added to the control group except the culture medium, and 10% dimethyl sulfoxide was added to the blank group (group 1).

### Statistics

Error bars were obtained applying the standard deviation function on three replicates. The data were statistically analyzed using SPSS 22.0 statistical software (SPSS Inc., USA). Student’s *t*-tests were performed and *P* values less than 0.05 was considered statistically significant.

## Results

The obtained ADM samples were examined by SEM

**Figure 1.**
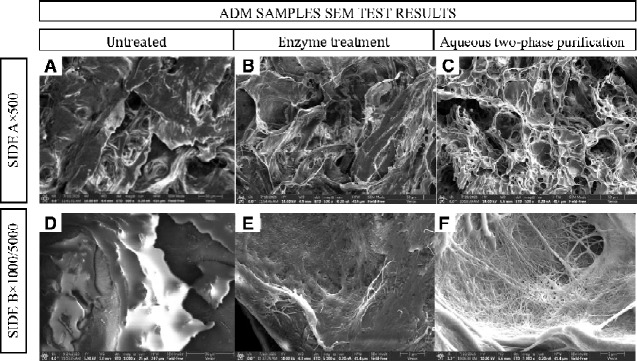
SEM Images of the three studied groups of ADM samples obtained with different sides untreated and prepared by papain and CPL (the ratio of papain and CPL was 50:50).

The SEM images obtained for untreated pigskin (control group), enzyme treatment and aqueous two-phase purification were analyzed. As shown in Fig. 1, SEM images obtained from decellularized tissues showed that the tissues have a hollow, tubular structure, unlike untreated tissues. This observation implied that most of the cells were still present in the untreated tissues, but had been removed from the decellularized tissues. In the images of the enzyme treatment group, numerous uneven distribution holes and irregular protein fibers had appeared. Concurrently, a small number of cells was still attached to the fiber clusters. By contrast, SEM images obtained for the aqueous two-phase purification groups featured clearly isolated cell holes, which are a characteristic structure of collagen fibrous interconnected networks formed as a result of ADM degradation was observed. Moreover, the collagen bundles were intact and distributed in an orderly manner. Additionally, the image of decellularized ADM depicted pores composed of tunnels of interconnected tubes in varied pore sizes ranging from 30 to 100 μm.

### Thermal stability of ADM samples

**Figure 2.**
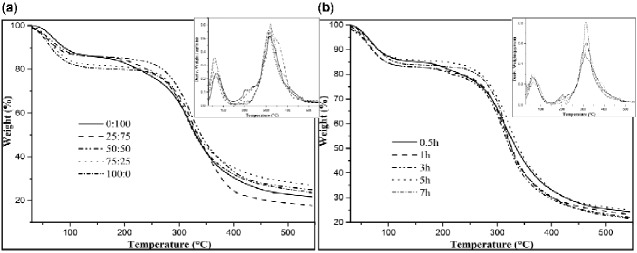
(**a**) Comparison among TGA thermograms of ADM samples from different enzyme content with the same enzyme treatment time (5 h); (**b**) comparison among TGA thermograms of ADM samples from different enzyme treatment times with the same enzyme ratio (50:50).

TGA was used to study the reaction thermal property of the ADM samples obtained from pigskin, and two separated weight loss procedures were observed in all thermograms (Fig. 2). The first step of analysis beginning at room temperature and ending at about 200°C was assigned to the loss of moisture. The second step reflected the volatilization of collagen fragments caused by thermally activated decomposition. Therefore, the starting temperature was regarded as the upper limit of thermal stability (T_dg_; Table 2). The temperature value of this limit was found to range between 193.2°C and 219.5°C, depending on the collagen structure and preparation. Meanwhile, we found that the T_dg_ values of ADM-14 and ADM-24 were 219.4°C and 219.5°C, respectively. However, they differed at 550°C, showing an approximately 3.12% difference in residue. As shown in Table 2, in the system composed of 50% papain and 50% CPL, T_dg_ and the residual amount of the material were relatively stable at around 210°C and 24%, respectively.

**Table 2.** Thermal performance of ADM samples 1–25

Study group	Enzyme	Hour (h)	T_d_ (°C)	**Δ**H_d_ (mj•g^−1^)	T_dg_ (°C)	TG residue (%)
ADM-1	Papain 0% CPL 100%	0.5	79.84	327.8	208.81	27.133
ADM-2		1	83.85	348.6	211.08	22.811
ADM-3		3	85.12	385.8	209.56	26.609
ADM-4		5	83.07	398.6	209.56	21.536
ADM-5		7	82.73	481.0	195.78	25.509
ADM-6	Papain 25% CPL 75%	0.5	85.15	324.4	206.78	15.672
ADM-7		1	87.12	439.9	209.12	15.374
ADM-8		3	83.17	483.2	210.32	22.41
ADM-9		5	78.79	239.1	214.10	17.88
ADM-10		7	75.01	497.8	214.86	21.84
ADM-11	Papain 50% CPL 50%	0.5	78.69	475.8	205.78	21.695
ADM-12		1	88.78	479.6	208.81	23.181
ADM-13		3	90.48	492.7	215.62	24.403
ADM-14		5	92.09	568.9	219.40	26.78
ADM-15		7	89.70	541.8	217.53	22.221
ADM-16	Papain 75% CPL 25%	0.5	78.38	522.4	202.75	23.622
ADM-17		1	87.57	446.4	215.62	24.226
ADM-18		3	84.72	549.9	217.13	22.852
ADM-19		5	85.75	497.9	216.38	27.105
ADM-20		7	82.54	279.2	193.20	17.790
ADM-21	Papain 100% CPL 0%	0.5	73.23	378.9	209.25	26.25
ADM-22		1	77.52	481.7	214.75	11.9
ADM-23		3	76.68	344.6	215.75	26.05
ADM-24		5	84.82	462.1	219.50	23.66
ADM-25		7	79.66	349.6	199.73	14.411

A comparison of the derivative plots (Vice map) of the thermograms for different ADM samples proved the occurrence of a main degradation process with a maximum peak in the derivative plot at 320°C. The ADM samples also exhibited a second peak at 60–70°C, while a minor peak was detected at 201–220°C, but the intensity of peaks differed. The divergence among the thermograms of samples for different preparation conditions was due to the degradation of the non-protein compound present in the samples. However, the changes in the foremost peak demonstrated some interaction between collagen and other ingredients, which affected the thermal conductivity of the collagen.

Collagen scaffold with comparable time but from different enzyme contents differed in terms of residues at 550°C. The proportion of enzymes of 25:75 and 75:25 displayed the lowest and highest values, respectively. Under the same enzyme content, the highest residuum could be obtained at 5 h. The residual amount was related to the type of protein and its preparation conditions to a certain extent, and the conversion temperature and material stability were positively correlated.

### DSC analysis of ADM samples

**Figure 3.**
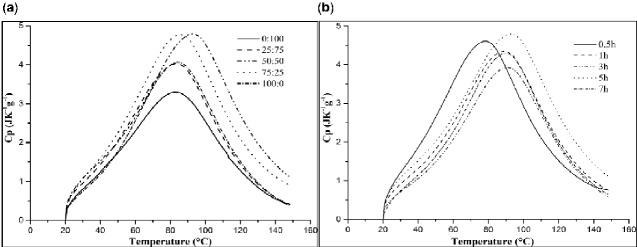
(**a**) Comparison among DSC analysis of ADM samples from different enzyme treatment times with the same enzyme ratio (50:50); (**b**) comparison among DSC analysis of ADM samples from different enzyme content with the same enzyme treatment time (5 h).

DSC analyses of all studied scaffold materials demonstrated the presence of a denaturation peak at comparatively high temperature: the maximum peak was recorded from 73.23 to 92.09°C and 78.69 to 92.09°C (Fig. 3). The shift in the maximum peak partly indicated that the degradation temperature of the materials was affected by the protein structure, further affecting its nature. ΔHd was calculated from the peak area of the maximum peak. The enthalpy transition reflects the degree of ordered structure of the globulins upon the conversion from natural state to denatured state. In addition, no correlation between ΔHd and Td was found. These findings indicated that the denaturation temperature was unrelated to the degree of structural order but it was related to the internal structure.

### FTIR spectroscopy analysis of ADM samples

**Figure 4.**
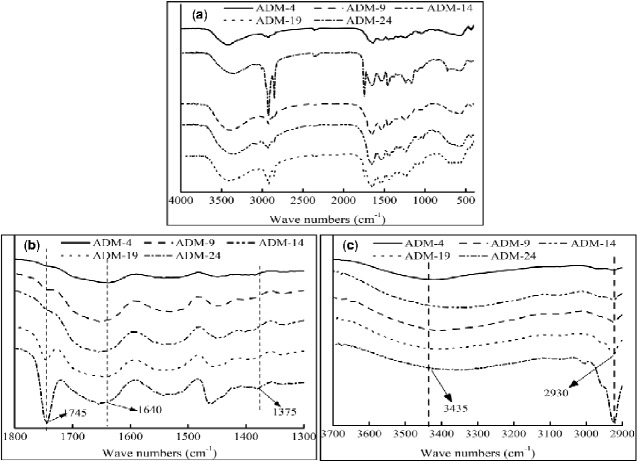
Comparison among FTIR of ADMs from different study groups. (**A**) is the original image, (**B**) and (**C**) are partial enlarged images of (A).

FTIR spectroscopy was conducted to study the possible compositional differences among the scaffold samples. As expected, the separated absorption bands were recorded in the FTIR spectrum (Fig. 4): amide I at 1640 cm^−1^ was caused by the C = O stretching vibration; amide II at 1539 cm^−1^ was due to the coupling of N–H in-plane bending and C–N stretching modes; amide III between 1090 and 1375 cm^−1^ region was attributed to C–N stretching and N–H bending. The absorption peak at 1463 cm^−1^ characterized the cis-configuration of the peptide bond, indicating that the collagen molecule contained a large amount of proline and hydroxyproline. In addition to the peptide units of proline residues, N–H and C = O of the peptide units in most polypeptide chains were arranged in trans, thereby indicating the integrity of the collagen structure of pigskin. Moreover, the absorption band at 3435 cm^−1^ was caused by the stretching vibration of O–H and N–H, indicating the existence of an admin A band. However, the absorption band at 2930 cm^−1^ was attributed to the stretching vibration of C–H, C–H_2_, and C–H_3_ of aliphatic compound. The band 2930 cm^−1^ was attributed to the presence of N–H and C–H groups, which demonstrated the existence of hydrogen bonds between the chain spiral chain. A comparison of the FTIR spectra of ADM samples at different conditions proved an increase in intensity of the absorption band at 2930 cm^−1^ with a decrease in the CPL content.

### Mechanical properties of the obtained ADM samples

**Figure 5.**
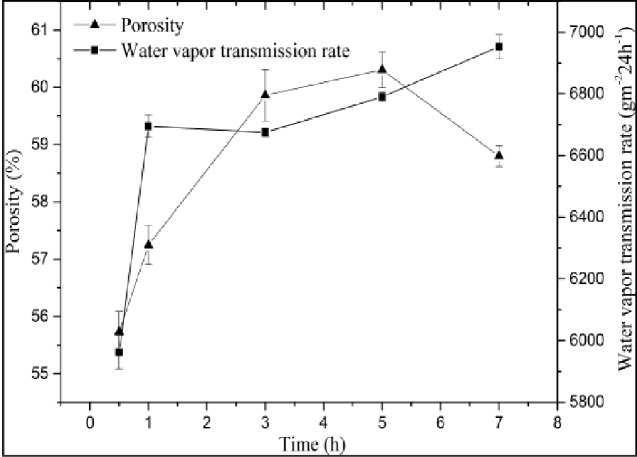
Comparison among mechanical properties of ADM samples from different times.

As shown in Fig. 5, we observed a porosity of 55.2% with the ethanol infiltration method under the shortest time, meeting the requirements for materials. Under increasing time, the porosity of the materials initially increased and then decreased, reaching a maximum of 60% or increase of 4.8%. This trend may be due to the decomposed cells and water over a long preparation of time; after the freeze-drying process, vapor bubbles took the shape of a large number of pores in the ADM samples due to sublimation. However, if the time was too long, then the collagen structure may be destroyed, resulting in the irregular appearance of the surface.

Under the same time conditions, the water vapor transmission rate was not significantly different between samples treated for a short time and relatively long time treated at any preparation conditions from 1 to 7 h. After the use of enzymes, the fibrous interstitium was fully hydrolyzed, and the collagen fibers were arranged more loosely.

### DNA quantification

**Figure 6.**
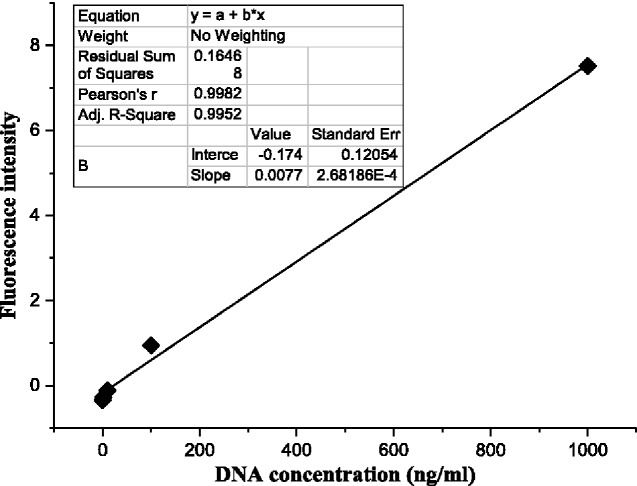
DNA standard curve.

**Figure 7.**
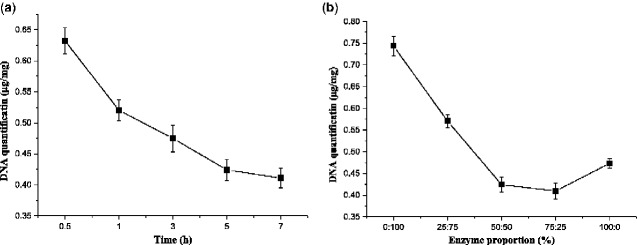
(a) Comparison among DNA content of ADM samples from different times with the same enzyme ratio (50:50) and (b) from different enzyme proportions with the same enzyme treatment time (5 h).

Quantitative DNA detection reflects the amount of nucleic acid residue in a material, and it was used as an evaluation index for the biocompatibility of the material. According to the instructions of PicoGreen fluorescent dyestuff, a standard curve was plotted with the DNA concentration and fluorescence intensity (Fig. 6). The DNA content of untreated pigskin was 2.16 ± 0.1 μg/mg dry weight, and it decreased from 0.63 ± 0.1 μg/mg dry weight at 0.5 h to 0.41 ± 0.1 μg/mg dry weight at 7 h (Fig. 7a). The longer the enzymolysis time, the looser the protein structure, and the more DNA double strands were exposed, and the better the effect. Meanwhile, the DNA content was 0.74 ± 0.1 μg/mg under 0:100 enzyme proportion compared with 0.47 ± 0.1 μg/mg under 100:0 enzyme proportion (Fig. 7b). The enzyme ratios of 50:50 and 75:25 showed low DNA contents, and the highest DNA contents were observed 0:100. This result indicated that papain could remove more cells than CPL.

### Hemocompatibility of ADM samples

**Figure 8.**
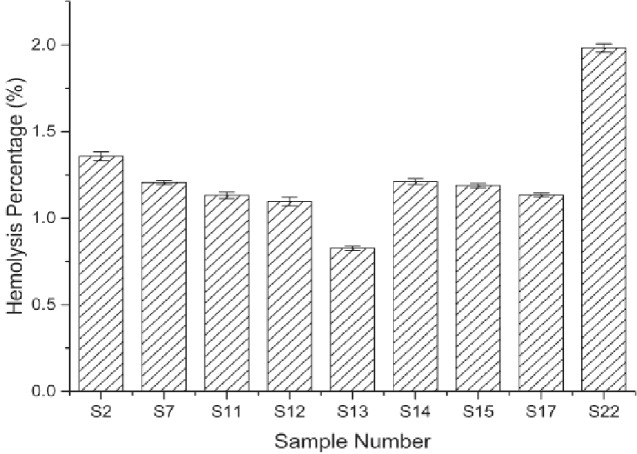
Comparison among hemolysis percentage of ADM samples from different sample numbers.

The contact of red blood cells with materials throughout their life cycle may significantly affect their inherent functions. Therefore, the hemocompatibility of ADM microparticles was assessed by hemolysis. The hemolysis percentage denotes the degree of red blood cells broken by the test sample in contact with blood. The results revealed that the hemolysis percentage was lower than 2% for ADM microparticles of different groups (Fig. 8), demonstrating good anti-hemolysis characteristic among all the samples.

### Cytocompatibility study

**Figure 9.**
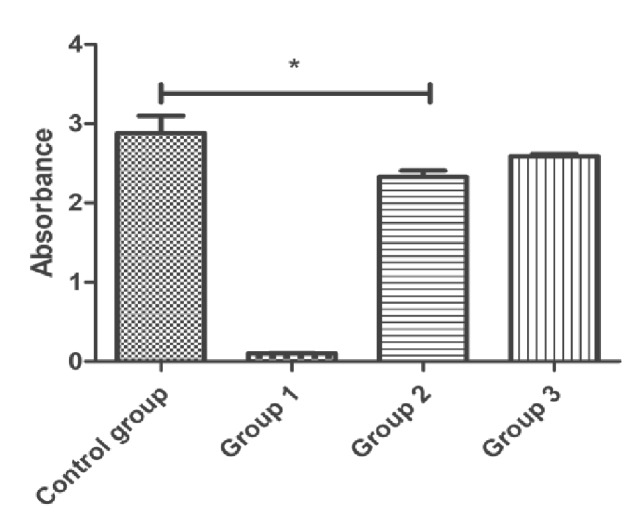
Comparison of absorbance of leaching solution after 72 h of cell culture. (group 1: negative control; group 2: 100% leaching solution; and group 3: 50% leaching solution). Statistical significance: *P < 0.05.

CCK-8 has been widely accepted as a characterization method for cell toxicity and proliferation. Cell viability (C_v_) was calculated with Equation (4). The optical density (OD), which indirectly represents cell viability, dropped to below 15% at 72 h (Fig. 9). Compared with the control group, the cell viability of the experimental group was 87% and 91.2%, respectively, which concluded that the ADM microparticles were not cytotoxic to the cells.

(4) Cv=(As-Ab)/(Ac-Ab)×100%

where As, Ac and Ab are the absorbance of the test samples, control group and blank group, respectively.

## Discussion

This work aimed to develop a plant enzyme-based collagen scaffold and investigate the potential to repair skin defects in clinical settings. After decellularization, the microstructure and composition of collagen fiber were very similar to human decellularized tissue. The ADM samples demonstrated that the enzymes could be used in removing fat tissue and a large number of cells in the epidermal structure without altering the mechanical properties of collage tissue. The maximum cell activity was maintained in the biological scaffold through this method.

DNA quantification demonstrated the presence of a small amount of DNA in the decellularized matrix. Compared with untreated pigskin, the DNA content changed significantly. An approximately 44.6% reduction in the DNA content of the decellularized tissue was observed, which could be due to the hydrolysis of histone associated with DNA. Thus, free DNA was shed from decellularized tissue. Papain is a cysteine protease that can degrade connective tissue proteins into small peptides and even amino acids.

SEM is usually recognized a useful tool for microstructural analysis [24, 25]. The SEM images of decellularized scaffold suggested that the number of holes increased and the overall structure of collagen was not destroyed in the matrix surface by the preparation process. This effect could be attributed to the natural properties of papain, which is recognized as an effective hydrolyzing protein that does not decompose collagen fibers [26]. To investigate the effect of using an aqueous two-phase system, the SEM images of the two different preparation processes were compared. This part of the study was performed in an aqueous two-phase system consisting of polymer and inorganic salts. The two substances were chosen for research because they are easy to phase, inexpensive and readily recycled. The results showed that the pores of the scaffold material were more obvious and evenly distributed by aqueous two-phase treatment, and collagen fiber clusters could be observed. The interconnected porous structure was beneficial for cell adhesion and proliferation. This phenomenon may be due to the free proteins and cells in the matrix, which showed high affinity for the polymer phase and preferentially partitioned to the PEG-rich phase by the separating phase. Meanwhile, the activity of most biological molecules was retained during two-phase treatment, and the substances formed by the two phases were non-toxic to the material [27].

**Figure 10.**
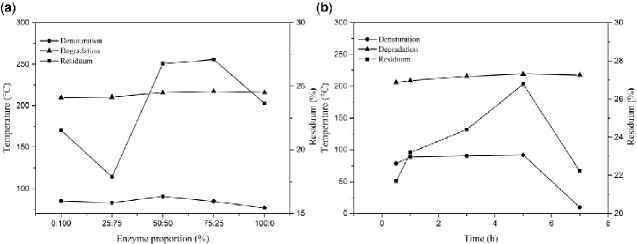
(a) Comparison among thermal properties of ADM samples from different enzyme content with the same enzyme treatment time (5 h) (b) and from different enzyme treatment times with the same enzyme ratio (50:50).

Thermal and spectroscopic results obtained by TGA, DCS and FTIR showed a correlation with the preparation conditions to determine the best method among the obtained data. In Fig. 10a, the correlation plots for the proportion of enzyme are reported, showing that the increase in papain proportion, from 0 to 50%, resulted in a significant increase in denaturation temperature of ADM samples, reaching the maximum value of 90.48°C. The denaturation temperature of ADM samples significantly decreased as the papain proportion exceeded 50%. By contrast, the degradation temperature was almost unaffected by enzyme variations and remained around 210°C for all samples. Collagen scaffolds with different enzyme contents had the largest residual amount under 50:50 and 75:25. In Fig. 10b, with the increase in the time, the denaturation temperature of ADM samples showed an upward tendency, with the maximum value of 92.09°C obtained at 5 h. At the same time, the degradation temperature of ADM samples followed a similar trend to the denaturation temperature. Collagen scaffolds with identical enzyme content but from different times differed in the residue at 550°C: 0.5 and 5 h displayed the lowest and highest values, respectively, with intermediate values for 1, 3 and 7 h. Studies have shown that pigskin collagen belongs to type I of collagen. Above 60°C, the hydrogen bonds between the original three helical chains of pigskin collagen are opened, and the helical structure of collagen is transformed into a random coiled structure, resulting in the denaturation of collagen. Thus, the compact fiber structure of ADM samples can increase its denaturation temperature to a certain extent. It may also be due to other non-protein co-products on the surface.

Further analysis of the infrared behavior revealed that amide I, amide II and amide III at 1638, 1539 and 1090–1350 cm^−1^, respectively, were caused by C = O stretching, N–H in-plane, and C–N stretching, respectively. These bands characterize the existence of the collagen triple helix structure and illustrate the structural integrity of the extracted pigskin collagen [28]. Meanwhile, the absorption bands of 2930 cm^−1^ showed different intensities among five groups: the peak of ADM-24 was the highest compared with the other samples, and it was attributed to the stretching vibration of C–H, C–H_2_ and C–H_3_ of aliphatic compound. This phenomenon may be explained by the fact that CPL was not used during preparation in ADM-24, indicating that CPL is an effective factor in removing fat from tissues.

## Conclusion

By comparing the results of thermal performance, spectroscopic characteristics, mechanical properties and DNA quantification, a preparation time of 5 h and enzyme content of 50:50 were better than others, and aqueous two-phase purification was a useful method for ADM preparation. By comparing DNA residue, hemolysis rate and cytotoxicity experiments, ADM prepared with plant enzymes demonstrated good biocompatibility.

## Funding

This work was supported by key scientific research project plan of colleges and universities in Hainan Province (RZ2000001667), Changzhou PARSD Biomedical Material Research Center (RH2000003058).

*Conflict of interest statement*. None declared.
